# Tumor Pre-Analytics in Molecular Pathology: Impact on Protein Expression and Analysis

**DOI:** 10.1007/s40139-018-0179-5

**Published:** 2018-09-06

**Authors:** Veronique M. Neumeister, Hartmut Juhl

**Affiliations:** Indivumed, GmbH, Falkenried 88, D-20251 Hamburg, Germany

**Keywords:** Tumor pre-analytics in precision medicine, Biospecimen processing and storage, Tissue integrity, Protein biomarkers as companion diagnostics

## Abstract

**Purpose of Review:**

Precision medicine promises patient tailored, individualized diagnosis and treatment of diseases and relies on clinical specimen integrity and accuracy of companion diagnostic testing. Therefore, pre-analytics, which are defined as the collection, processing, and storage of clinical specimens, are critically important to enable optimal diagnostics, molecular profiling, and clinical decision-making around harvested specimens. This review article discusses the impact of tumor pre-analytics on molecular pathology focusing on biospecimen protein expression and analysis.

**Recent Findings:**

Due to busy clinical schedules and workflows that have been established for many years and to lack of standardization and limited assessment tools to quantify variability in pre-analytical processing, the effects of pre-analytics on biospecimen integrity are often overlooked. Several studies have recently emphasized an emerging crisis in science and reproducibility of results.

**Summary:**

Biomarker instability due to pre-analytical variables affects comprehensive analysis and molecular phenotyping of patients’ tissue. This problematic emphasizes the critical need for standardized protocols and technologies to be applied in the clinical and research setting.

## Introduction

Efforts are underway to better understand tissue phenotypes and molecular characteristics of individual patients aiming to provide a personalized medicine approach to deliver timely and targeted prevention and treatment [1]. As technological advances facilitate comprehensive analyses of possible drug targets at affordable costs, this vision of personalized and patient tailored medicine is a driver for development of individualized therapeutic strategies and companion diagnostic tests to accurately stratify and treat patients. Throughout this clinical development, biological assays and assessment of biomarkers play an important role in providing diagnostic, prognostic, and predictive information [2, 3]. However, various limitations and challenges need to be considered when translating promising biomarkers and drugs to the patient. A number of efforts and clinical trials have failed [4, 5], with confounding issues being limited knowledge of analytical, diagnostic, and regulatory requirements for clinical assays as well as lack of standardization on various levels. These issues extend beyond the ones listed with variability and lack of standardization in pre-analytical processing being often overlooked. Pre-analytical variables include, but are not limited to, anesthesia, surgical procedures, warm and cold ischemic time, tissue processing, fixation, and storage of biospecimens.

Here, we aim to discuss and review the effects of tumor pre-analytics on protein expression resulting in modification of in vivo protein status.

## Anesthesia and Surgical Approaches

### Anesthesia

Several publications describe the influence of anesthetic drugs and methods on tumor biology and alterations of biomarkers. Effects of general anesthesia on blood and serum biomarkers were investigated in a few studies showing that most of the tested analytes were not affected by time of blood draw or administration of anesthesia [6, 7], while viability and apoptosis of circulating CD4-positive lymphocytes were significantly affected by propofol administration [8]. No changes were reported for circulating CD8-positive lymphocytes. Metabolomic profiling of pre- and postanesthesia plasma samples of colorectal cancer patients revealed that propofol- or etomidate-induced anesthesia significantly decreases levels of several metabolites compared to pre-anesthesia blood draw [9]. With an increasing number of highly sensitive biomarkers and multi-analyte blood tests, the pre-analytical variability of liquid biopsies introduced by administration of general anesthesia should still be taken into consideration. Several publications also report potential links between the administration of analgetics, anesthetics, and molecular changes in tumor biology [10–12]. Opioid therapy, for example, directly activates μ-opioid receptor (MOR) expression in cancer cells. Overexpression and activation of MORs promote activation of Akt and mTor, and MOR agonists may influence expression levels of these important biomarkers [13]. MOR also interferes and transactivates VEGFRs in cancer cells [14], as well as EGFR phosphorylation, AKT, and MAPK/ERK activation [15]. MOR plays an important role in NSCLC and has reportedly been associated with NSCLC progression and metastasis [11, 16]. Influence of general anesthesia on metastatic potential of cancer cells, recurrence, and overall survival was studied in several cancer types, such as colon, breast, and prostate cancer [17–20]. These observations might not only be associated with direct activation and interaction of MOR and opioid agonists with cancer cell receptors and pathways. Opioids and other anesthetic drugs, such as benzodiazepine derivatives, propofol, ketamine, and others, are also directly linked to immune-suppression and have immune-modulatory effects [21]. These range from inhibition of transcription factors that regulate production of inflammatory mediators, to direct interference with receptors on macrophages, to central neuro-endocrine/neuro-paracrine and peripheral mechanisms, and to peripheral actions mediated by mu-opioid receptors on immune cells [22]. While immune-suppressive effects of anesthesia and transient immune-impairment seem to primarily affect the general immune system of the patient, immune-modulatory influences on the tumor environment, tumor infiltrating lymphocytes, and alterations in NK cell activity may enhance tumor growth and metastatic ability and alter matrix metalloproteinase and MOR status within the tumor [23–25]. Despite all this evidence, data on the influence of general anesthesia and even cancer progression are still controversial, and the substantiation is insufficient to support any changes in current clinical practices [26]. In an attempt to reduce intraoperative opioid consumption and surgical stress response [27, 28] and improve postoperative pain management, epidural or regional anesthesia can be combined with general anesthetics. Experimental data from various animal models suggest that regional anesthesia attenuates the process of metastasis by preserving natural-killer (NK) cell function and modification of the T-lymphocytic population [24, 29, 30]. Different studies in patients undergoing surgery for breast, lung, and other cancers have shown that the combination of epidural with general anesthesia results in a significant increase of CD8-positive lymphocytes within the tumor microenvironment but a decrease in FOXP3-positive T cell infiltration [24, 30, 31], a preservation of NK cells and of the preoperative balance of the patient’s immune system and tumor microenvironment [32]. These results suggest that combined epidural and general anesthesia mitigates the suppression of immune functions caused by surgical stress and various analgetics/anesthetics and improves pain management and postoperative recovery.

### Surgical Approach and Warm Ischemia

Not only do methods of anesthesia and surgical stress attenuate the patient’s immune system, have immune-modulatory effects within the tumor microenvironment, and modify several receptors and downstream pathways, the surgical approach, duration of it, and time to tissue removal also significantly impact the molecular tumor phenotype. The most important factor herein is warm ischemic time, defined as the time a tissue, organ, or body part remains at body temperature after its blood supply has been reduced or cut off but before it is cooled, further processed, or reconnected to a blood supply. The extent of warm ischemic time depends on the procedure and organ, the surgical approach with laparoscopic surgeries sometimes doubling or tripling warm ischemia [33], the experience of the surgeon, and other confounding factors. Tissue integrity and histopathological characteristics remain mainly unaffected leaving the tissue suitable for assessment of pathological stage, grade, and further evaluation [33, 34], whereas hypoxia and stress induce tumor cell responses on a genetic, transcriptome, and protein level. The effects of warm and cold ischemia can be classified into ischemia-induced metabolic responses including posttranslational modification, all of which occur within the early stages after ligation of the blood supply, and ischemia-induced degradation on a cellular and tissue level as a result of hypoxia and stress [35]. Furthermore, these metabolic responses, posttranslational modifications, and degradative processes are highly variable comparing normal and tumor tissue, with malignant tissue having significantly higher variability and reactions to stress and hypoxia [36]. Additional variability lies within different organs and patient populations. Comparing molecular tumor phenotypes from surgical biopsies before and after clamping of the main blood vessels and removal of the tissue reveals that the severity of posttranslational modification and stress response and the number of involved genes and proteins increase with prolonged ischemic time [36]. Several important biomarkers and therapeutic targets such as mTOR, ERK1/2, AKT, and MEK are upregulated within 10 min of warm ischemic time, while their expression levels subsequently decrease again. Some proteins such as EGFR reveal high interpatient variability in their response to pre-analytical differences in tissue acquisition. Most vulnerable and reactive are phosphorylated proteins [37], and the phosphorylation status of key signaling proteins is significantly altered within a short period of ischemic time both in normal and in tumor tissue samples [36]. While most proteins show a decrease in phosphorylation, markers of posttranslational modification and stress response increase gradually. With warm ischemic time being dependent on the surgical procedure, anatomy, patients’ factors, and other influences, standardization, conclusions, and prospective studies are difficult to attain. While it is hard to minimize and control warm ischemic time, documentation of it is helpful in determining a degree of stress and changes within the molecular phenotype of biospecimens that might have occurred.

## PostSurgical Factors Confounding Biospecimen Quality

A variety of factors involved in tissue handling and processing such as cold ischemic time, fixation processes, storage conditions, and others lack standardization and guidelines, all of which have potential influence on clinically relevant target molecules and biomarkers.

### Cold Ischemia

**Fig. 1:**
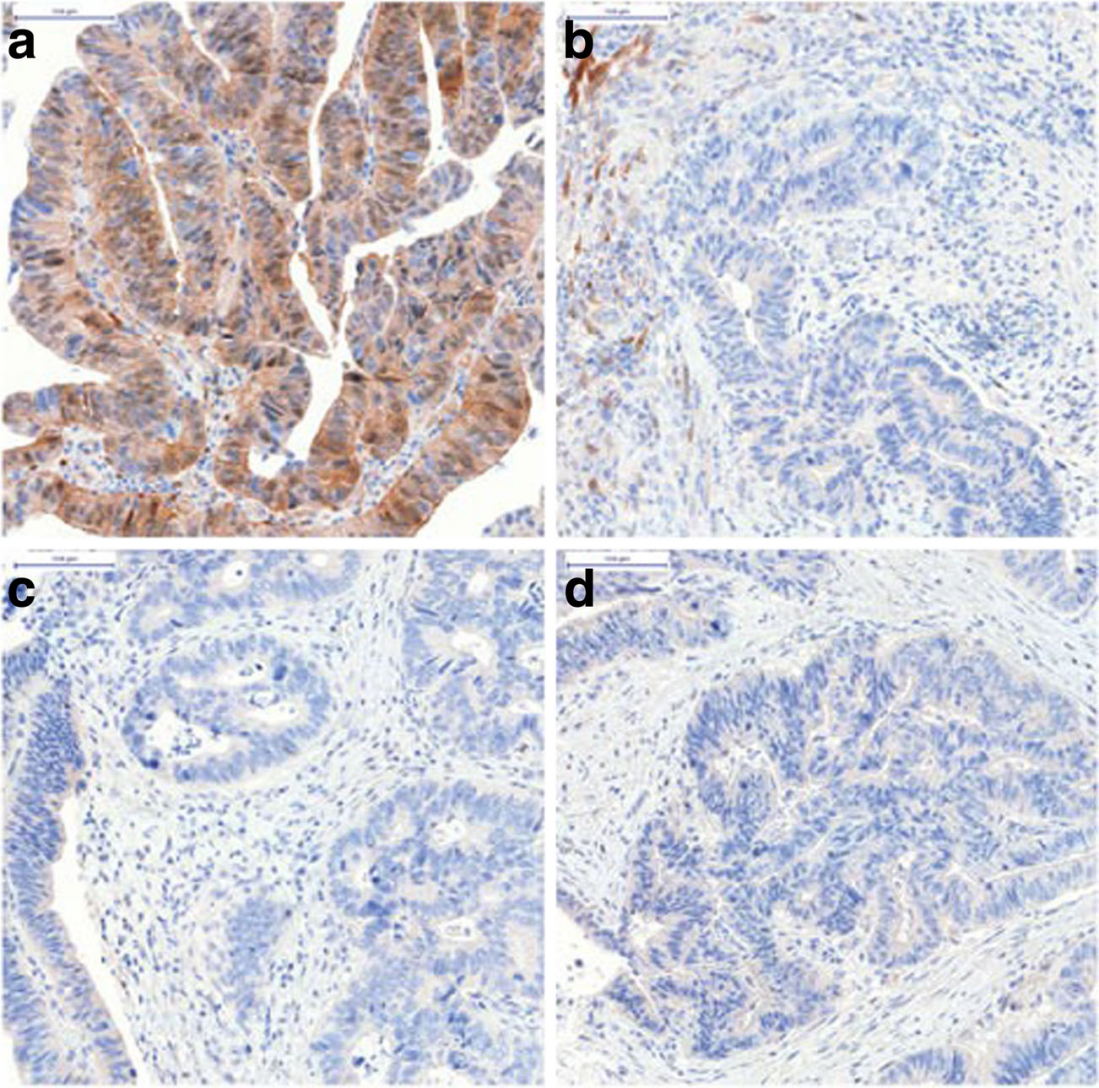
Representative images illustrating significant reduction in expression levels of phospho-AKT according to increasing cold ischemic time ([36]). These images show evaluation of pAKT expression by immunohistochemistry on formalin-fixed colon cancer tissue from one patient taken at four timepoints. **a** Biopsy presurgery; **b** Tissue fixed 10 min after resection; **c** Tissue fixed 20 min after resection; and **d** Tissue fixed 45 min after resection

**Fig. 2:**
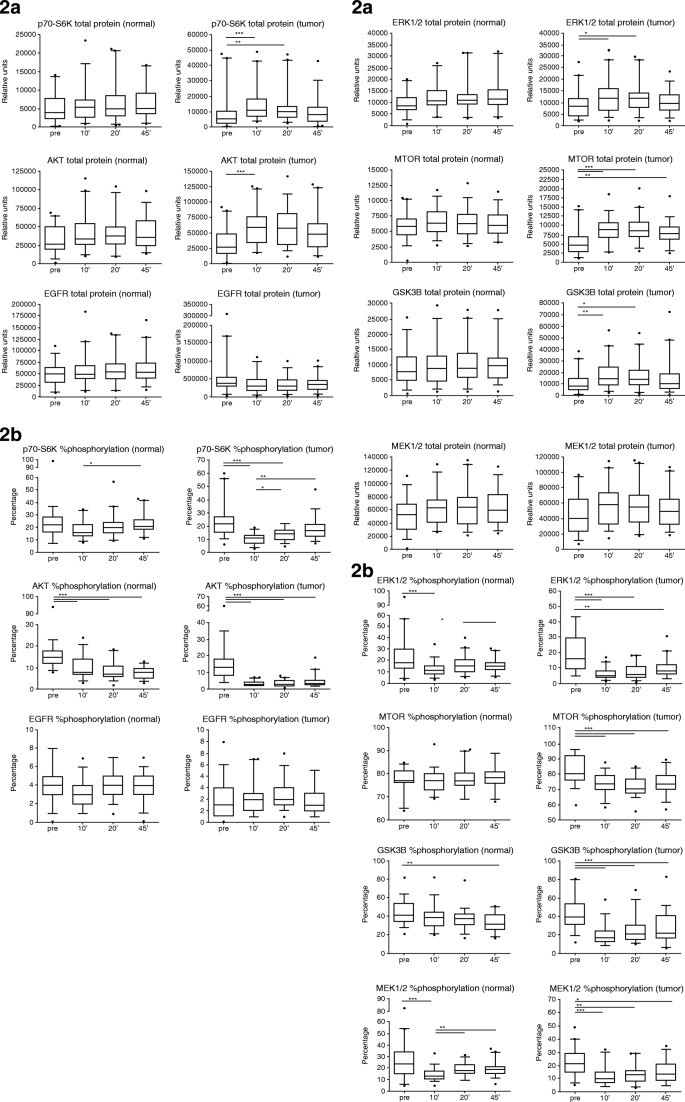
Examples of changes of protein expression levels according to increasing cold ischemic time ([36]). **a** Total protein expression (relative units) of p70-S6K, AKT, EGFR, ERK1/2, MTOR, GSK3B, and MEK1/2 in normal and tumor colon tissue at four timepoints of tissue collection: pre, before hepatic pedicle clamping; 10′, 10 min after resection; 20′, 20 min after resection; and 45′, 45 min after resection.*p < 0.05; **p < 0.01; ***p < 0.001. Box plots indicate the 5/95% confidence interval, median, and standard deviation. **b** Percentage of protein phosphorylation of p70-S6K, AKT, EGFR, ERK1/2, MTOR, GSK3B, and MEK1/2 in normal and tumor colon tissue at four timepoints of tissue collection: pre, before hepatic pedicle clamping; 10′, 10 min after resection; 20′, 20 min after resection; and 45′, 45 min after resection. *p < 0.05; **p < 0.01; ***p < 0.001. Box plots indicate the 5/95% confidence interval, median, and standard deviation

The common definition for cold ischemia in surgery is the time between the chilling of a tissue, organ, or body part after its blood supply has been reduced or cut off until it is warmed by having its blood supply restored. In biospecimen science, cold ischemic time is defined as the period of removal of an organ/tissue/biospecimen from the body until further preservation of the specimen such as chemical fixation or snap freezing. While reports on the effects of warm ischemic time are scarce, several publications investigate molecular changes of patients’ samples that are attributed to delay in tissue processing and archiving after it was harvested. Cold ischemia triggers a cascade of noxious effects and responses to hypoxia and stress, all of which affect the quality of the biospecimen and subsequently of any analytical approach [38–43]. Comparing molecular changes at various timepoints of cold ischemia reveals that already 15 min after surgery, a certain percentage of detectable genes and proteins (with reports ranging from 1 to 15%) and 30 min after surgery up to 20% of all detectable molecules show moderate to significant changes from baseline values [44–48]. Variability in tissue response is not only confounded by tissue, patient, and population heterogeneity [49–51] but also by the complexity of phosphorylation cascades of different proteins and isoforms, hypoxia-induced responses, posttranslational modification, degradation, and increased vulnerability of tumor compared to normal tissue. The complexity of all these factors results consequently in a fluctuation of expression levels of phosphorylated proteins within the first 20 to 30 min of cold ischemic time [37, 52•], followed by dephosphorylation processes within 1 to 2 h of delay to formalin fixation leading to a significant decrease to loss of the majority of phospho-epitopes [45, 53–60]. Examples for more vulnerable phospho-epitopes are p-AKT, p-MAPK, p-Tyrosines, and p-Met amongst others, all of which are important biomarkers for pathway activity and actionable drug targets (Fig. 1). While the phosphorylation status of several proteins is altered significantly within a short period of time, the non-phosphorylated molecules remain more stable [37]. To complicate matters, other phosphorylated epitopes, such as p-ER, p-HER2, and p-Jak2, to name a few, are not as vulnerable and may remain stable in their expression levels for up to a few hours of cold ischemia [61]. Figure 2 illustrates variability in protein expression due to increasing cold ischemic time. The complexity of these results is also further confined by tissue and tumor heterogeneity [58], the method of assessment of the protein status (qualitative versus semi-quantitative and quantitative measurements), availability of appropriate antibodies to various isoforms, and validation of those. Moreover, these studies are not comprehensively evaluating the stability of protein phosphorylation over an entire sample population, but rather produce short time point data on a limited set of samples. Wu et al. describe the Bayesian model, a statistical approach to generate trajectories and estimates for the phosphorylation time course of various phospho-tyrosines on limited sample sets simulating larger sample sizes and cohorts. This model [62] computes similar results to the ones reported with fluctuation of phosphorylation and increases and decreases of phosphorylation abundance of phospho-tyrosines in a time-dependent manner following cold ischemic shock.

Due to post-excisional reactivity, dynamic state and adaptive cellular mechanisms in response to stress, hypoxia, and other environmental factors, certain proteins such as phospho-HSP27, a small heat-shock-induced protein and apoptosis modulator, acetylated lysin, a marker of posttranslational modification, AKAP13 and HIF1 alpha, and hypoxia-induced analytes are examples of proteins that show a proportional increase as a function of delayed time to fixation up to a period of 5 h [61]. Standard breast cancer biomarkers on the other hand, such as estrogen receptor (ER), progesterone receptor (PR), HER2, and Ki67, are not affected by cold ischemic time of 1 h [61, 63], which is the maximum of delay to fixation for breast cancer specimens recommended by ASCO/CAP guidelines [64–66]. These proteins do reveal loss of antigenicity if cold ischemia extends beyond several hours impacting companion diagnostic testing and treatment decisions [61, 63, 67–69]. Lability of protein-biomarkers extends beyond these listed here, and a number of prognostic and predictive analytes in different tumor and tissue types are affected by extended cold ischemic shock confounding tissue quality [36, 60, 61, 70]. While several publications investigate protein expression levels using the binary qualitative evaluation of IHC, these results were also confirmed with quantitative approaches such as in situ quantitative immune-fluorescence, NanoPro 1000 technology—a quantitative immune assay platform—mass spectrometry, and reverse phase protein arrays [36, 52•, 60, 61].

In an effort to minimize detrimental effects of delay to tissue fixation, several centers keep biospecimens at 4 °C until further processing [71]. Studies have proven that chilling of the samples mitigates hypoxia-induced responses and degenerative effects for up to several hours of cold ischemia thus resulting in better preservation of tissue morphology, epitope stability—including phosphorylated proteins—and RNA integrity [60, 63, 72]. Gianni Bussolati et al. introduced the method of immediately vacuum sealing resected samples followed by storage at 4 °C up to 72 h as a feasible way to preserve biospecimens for an extended period of time [73–76], though it is not all clear if vacuum sealing renders additional value in tissue preservation or if cooling alone suffices [75].

### Fixation

Formalin fixation and paraffin embedding (FFPE) of tissue samples is a widely established and inexpensive method to process and archive biospecimens over long time periods. Several factors such as concentration, pH, presence/absence of buffer in the formalin solution, tissue-to-fixative-volume ratio, size and grossing of the tissue, temperature during and duration of the fixation process, postfixation processing, and paraffin impregnation need to be optimized and standardized to optimally preserve histomorphology and molecular phenotypes of harvested specimens [77–79]. Recommendations and guidelines advise fixation using 10% neutral buffered formalin (NBF) [64–66], though optimal preservation of different molecules might require different buffers or fixatives [78, 79]. Pre-analytical variability around formalin fixation and paraffin embedding and the problematic of suboptimal processing conditions have been summarized by Helen Moore and colleagues [77, 80]. In an attempt to improve preservation of several downstream targets and phospho-protein abundance while maintaining histomorphology in tissue blocks, several alternative fixatives were investigated, such as a one stop biomarker and histology preservative (BHP) consisting of reversible cross linkers, permeation enhancer, phosphatase and kinase inhibitors, and the fixative [81]. The Paxgene Tissue System represents another formalin-free tissue preservation technology that reportedly allows preservation of histomorphology, intact and immune-reactive proteins, and antigenicity, while also maintaining integrity of nucleic acids [82–84]. Signal intensities of a number of phosphorylated proteins preserved in Paxgene-fixed paraffin embedded (PFPE) specimens were comparable to those analyzed in cryopreserved samples, whereas expression levels of the same phospho-epitopes obtained from FFPE tissue samples were significantly weaker [85]. Prolonged duration of Paxgene fixation also did not negatively affect expression levels of analytes. These are just two examples of several attempts during the past years to replace cross-linking formalin with alternative preservatives aiming to improve the quality of proteomic and other molecular analytical approaches [86–89].

Snap freezing biospecimens is superior to chemical fixation. Preservation at ultra-low temperatures rapidly and effectively inactivates a broad range of protein modifying and processing enzymes and is also the method of choice for preservation of nucleic acids. Large-scale proteomic analysis including comprehensive phospho-proteomics yields significantly better results using snap frozen specimens.

### Long-Term Storage

The value of FFPE tissue, archives collected often over decades and huge biorepositories, is undisputed. Despite the fact that FFPE blocks are not the best source for highest quality molecular material, recent advances in extraction of nucleic acids and proteins have made these derivatives available to high throughput genomic and proteomic platforms. At the same time, the quality of FFPE biospecimens, often stored over a long-term period, needs to be critically evaluated to determine their fit-for-purpose for accurate molecular phenotyping. While formalin fixation and paraffin embedding preserves tissue morphology for up to 30 years of storage [90], including cytological details and immune-reactivity of tissue antigens, recent studies investigate the influence of long-term storage on complex molecular analytes. Results, however, are somehow controversial with studies concluding that the fitness of FFPE blocks for proteomic analysis is independent of tissue age evaluating a time frame of 11 years [91], respectively 11-year intervals from 1990 to 2001 and from 2002 to 2013 [92], but rather dependent on tissue and tumor type, reporting significant differences in protein derivatives between papillary, squamous, and adenocarcinomas as measured by protein absorbance values in one of the studies [92]. Comparing analysis of corresponding fresh frozen and FFPE tissue samples by multidimensional liquid chromatography-based mass spectrometry (LC-MS), the data generated indicate essential equivalence between protein inventories obtained from either source of processed and stored tissue [93]. Researchers conclude that the storage of FFPE blocks of roughly 10 years duration should not be a significant impediment to proteomic analysis. Very few studies investigate the influence of extended storage time. Combs et al. quantitatively analyzed expression levels of ER, Her2, Ki67, and cytokeratin (CK) on a series of FFPE tissues from more than 1000 patient samples preserved for 7 to up to more than 50 years. While the average expression decreased for all biomarkers over time, the rate of loss of antigenicity is target-specific [94•]. The results indicate a 10% loss of antigenicity for ER expression over a period of 10 years, while HER2 and Ki67 seem to degenerate faster with 10% loss of expression over a period of 8.9 and 4.5 years, respectively. The degradation of CK occurs at a much lower rate. While these results are restricted to four biomarkers in breast cancer specimens only, this study demonstrates that tissue age is an important variable and should be considered when investigating archived tumor samples. Several investigations have also addressed loss of protein expression in tissue sections that were stored over an extended period of time due to effects of air, humidity, temperature, and fixatives, leading to oxidation, denaturation, and further degradative modification of tissue biomarkers [71, 95–97].

## Conclusions

The concept of precision medicine, in which health care is tailored to each patient based on a person’s genes, lifestyle, and environmental factors, promises an opportunity to make precise personalized patient care a clinical reality. With this promise, there is critical need for standardized protocols and technologies that can be used in the clinical setting for seamless collection and preservation of biospecimens used for clinical decision-making. For whole genome arrays and comprehensive genomic profiling variations due to pre-analytical noise may be compensated by the large number of analyzed transcriptomes and non-coding regions. However, in case of validation of signatures, targeted sequencing, and foremost protein-based diagnostic tests that are the source of treatment decisions, the problem of biomarker instability and variability in pre-analytical tissue processing and storage is acute. The estimated number of papers documenting biomarker discoveries lies above 100,000, while the estimated number of biomarkers used in the daily clinical setting lies around 100 [98]. Lack of validation and qualification of biomarker-based research impacts product and drug development with pharmaceutical companies spending billions of dollars in drug discovery and pre-clinical and clinical phases, with the main expense being failure within these processes. Scientists at the biotech company Amgen reported that out of 53 landmark studies, only 6 could be proven valid raising concerns about an emerging crisis in science. Similarly, a group at Bayer HealthCare found that only 25% of publications, on which the company was basing R&D efforts, could be validated. While irreproducibility may partly result from trial and error inherent to the scientific process, the major problem lies within sample procurement, storage, analysis, and lack of standardization of these processes [5].

To date, clinical processing and preservation techniques rely mainly on protocols and approaches that are decades old, and in a busy clinical setting, improvement of processes is difficult. Therefore, unambiguous determination of in vivo levels of proteins is a challenge, and different protocols for tissue handling and protein preparation may result in significantly different protein profiles. NCI’s Biorepositories and Biospecimen Research Branch (BBRB) has found current tissue sample-handling techniques to be a major roadblock to future quality research and personalized medicine [99]. To improve collection and storage procedures and standardize practices across different institutions, the BBRB has issued the NCI Best Practices incorporating key principles to define state-of-the-science biospecimen resource practices, promote biospecimen and data quality, and support adherence to ethical and legal requirements, https://biospecimens.cancer.gov/practices/. The International Society for Biological and Environmental Repositories (ISBER) also published ISBER Best Practices: Recommendations for Repositories—evidence-based or consensus-based practices for collection, long-term storage, retrieval, and distribution of specimens. The College of American Pathologists (CAP) has published guidelines for breast cancer to standardize and optimize tissue acquisition, processing, handling, and testing [64, 65]. These guidelines comprise mandatory elements aiming to improve and standardize all aspects of companion diagnostic testing in breast cancer. Adherence to the NCI and ISBER Best Practices, however, is strictly on a voluntary basis. Additional important steps toward improving and standardizing biospecimen collection, processing, and quality are currently on their way. The Personal Healthcare Committee (PHC) of CAP initiated the formation of the Pre-analytics for Precision Medicine Project Team (PPMPT), a task force developing practice metrics and documentation guidelines to be applied in CAP-accredited laboratories as well as guidelines for the control of cold ischemic time and additional pre-analytical variables.

With increasing recognition of the importance of biospecimen quality for precision medicine, these initiatives, task forces, and guidelines are steps toward the goal to ensure that all biospecimens in research and clinical settings are fit for molecular analysis and represent the patient’s in vivo molecular profile.
